# YSZ-Reinforced Alumina Multi-Channel Capillary Membranes for Micro-Filtration

**DOI:** 10.3390/membranes6010005

**Published:** 2015-12-30

**Authors:** Bo Wang, Melanie Lee, Kang Li

**Affiliations:** Department of Chemical Engineering, Imperial College London, London SW7 2AZ, UK; bo.wang2@imperial.ac.uk; melanie.lee08@imperial.ac.uk

**Keywords:** ceramic membranes, YSZ, alumina, multi-channel, phase inversion

## Abstract

The combined phase-inversion and sintering method not only produces ceramic hollow fibre membranes with much lower fabrication costs than conventional methods, but these membranes can also be designed to have greatly reduced transport resistances for filtration processes. The bottleneck of this technique is the weak mechanical property of the fibres, due to the small dimensions and the brittle nature of the ceramic materials. In this study, yttrium stabilised zirconia (YSZ) reinforced alumina seven-channel capillary microfiltration membranes were prepared with a pore size of ~230 nm and their mechanical property and permeation characteristics were studied. It is found that the addition of YSZ can effectively enhance the mechanical property of the membrane and also increase pure water permeation flux. The Al_2_O_3_-YSZ seven-channel capillary membranes could reach a fracture load of 23.4 N and a bending extension of 0.54 mm when being tested with a 6 cm span, to meet the requirements for most industrial microfiltration applications.

## 1. Introduction

Ceramic hollow fibre membranes prepared by a combined phase-inversion and sintering technique have been intensively studied in the past decade [1]. In principle, the method uses a suspension containing ceramic powder, a polymer binder and a solvent that dissolves the polymer binder. Because of the good fluidity of the suspension, it can be easily extruded or casted. Upon contact with a coagulant that is miscible with the solvent but precipitates the polymer, the suspension loses the solvent and the polymer precipitates, so that green bodies composed of the ceramic powder and the polymer binder are obtained. The method has been particularly applied to make ceramic or metal micro-tubes with outer diameter from hundred micrometers to a few millimeters, which are usually called hollow fibers. Compared with traditional extrusion processes, the phase-inversion method can be used to prepare much smaller tubes, while capital investment is much lower. In addition, the phase-inversion method allows manipulation of the tubes’ wall structures, forming structures from highly asymmetric and porous, to symmetric and dense, offering more options to incorporate specific functions into the tube [2,3]. Though the process has not been widely used in the industry, it has shown potential in applications, such as membrane separation, membrane reactors and solid oxide fuel cells [4,5,6,7]. 

Prior to industrialisation of the ceramic hollow fibres, there are still problems that need to be solved, especially the mechanical property of the fibres. In general, the fracture load of ceramic hollow fibres is weak, not only due to the small dimensions of the fibres but also because of the presence of micro-channels formed in the fibres during fabrication. Although these micro-channels provide extra benefits for mass transport and functionalization, they also compromise the fibres’ mechanical property. One effective way to enhance the fracture load of the ceramic hollow fibre is to increase the cross section area by using multi-channel configurations. Our previous study showed that by applying multi-channel configurations to alumina micro-filtration hollow fibre/capillary membranes, not only the fracture load can be substantially enhanced, but also the apparent water permeation rate per unit out surface area can be increased due to the increase of effective permeation area [8]. It is however still not sufficient for these alumina membranes to be used in industrial filtration processes, where high flow rate and vigorous vibrations may lead to the generation of cracks in the fibres, especially under the impact produced at the startup or shutdown of the filtration plant.

To further improve the fracture load of ceramic hollow fibres and its mechanical stability under impacts, another approach is to enhance the mechanical strength of the membrane material. Strengthening the membrane material with an enforcing agent has been widely used in composite materials. A typical example is zirconia toughened alumina (ZTA) or YSZ toughened alumina [9]. In this research, 3 mol.% yttrium stabilized zirconia (3-YSZ) was used to reinforced the alumina material. By combining the advantages of multi-channel configuration, ceramic capillary membranes that can meet the mechanical requirements for use in industrial applications were produced. Furthermore, our study showed that the use of YSZ does not change the effective pore size of the membrane or compromise the water permeation flux. 

## 2. Results and Discussion

Seven-channel Al_2_O_3_-YSZ capillary membranes were prepared by using the combined phase-inversion and sintering process. The Al_2_O_3_-YSZ membrane material is composed of 30 vol.% 3-YSZ and 70 vol.% alumina.

### 2.1. Membrane Morphology 

**Figure 1:**
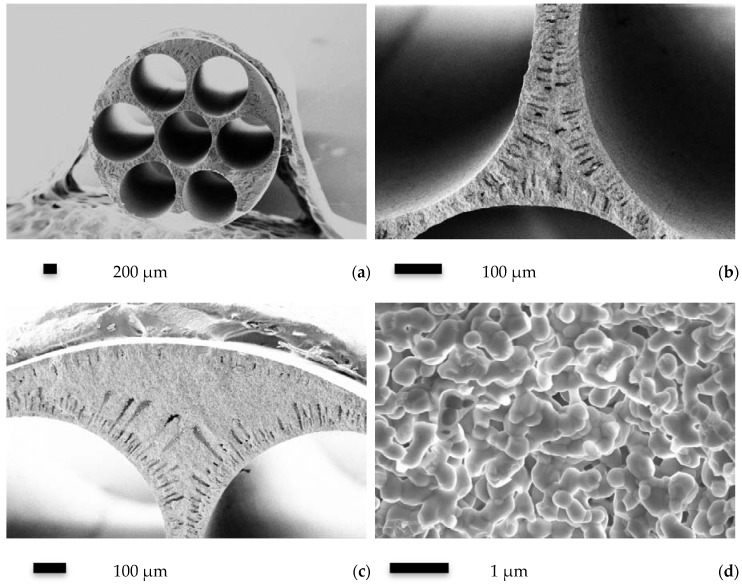
Microstructure of the Al_2_O_3_-YSZ seven-channel capillary membrane sintered at 1400 °C: (**a**) whole view of the cross section; (**b**) cross-sectional view of the internal wall between channels; (**c**) cross-sectional view of the outer wall and (**d**) micro-structure in the sponge-like region.

Figure 1 shows cross-sectional structure of the seven-channel Al_2_O_3_-YSZ capillary membrane sintered at 1400 °C. The membrane has a symmetric macrostructure with six channels distributing evenly around the central channel. The outer diameter is 3.2 mm and the inner diameter of each channel is around 1.0 mm. The walls between channels are composed of micro-channels and densely packed sponge-like regions. At the walls next to the outer surface, similar features are also present, but the micro-channels from the outer surface are much shorter than the micro-channels from the inner surfaces. The generation of micro-channels is due to interfacial instability and has been well explained elsewhere [10]. Closer examination on the microstructure of the cross section at the sponge-like part reveals the porous structure of the sintered sample, in which necks between ceramic grains can be clearly seen, indicating that proper sintering has been reached at 1400 °C.

**Figure 2:**
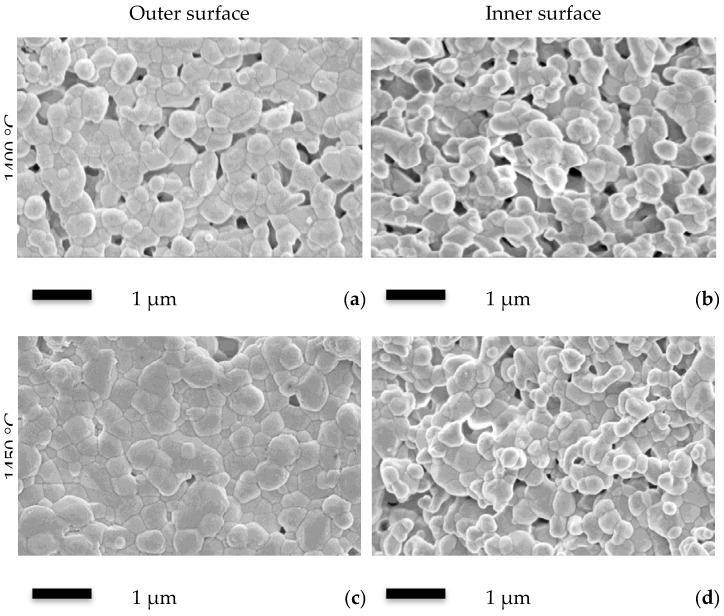
Outer and inner surface SEM images of the Al_2_O_3_-YSZ seven-channel capillary membranes sintered at 1400 and 1450 °C. (**a**) outer surface of the 1400 °C sample; (**b**) inner surface of the 1400 °C sample; (**c**) outer surface of the 1450 °C sample and (**d**) inner surface of the 1400 °C sample.

Figure 2 shows SEM images of the sintered membrane. Obviously, the outer surface was smoother than the inner surface, due to the fact that a short air gap was used during the spinning process, which gives a relaxation time for the outer surface to ease the disturbance produced by the spinneret. However, this relaxation was not possible at the inner surface, thus leading to the rougher surface (Figure 2a,b). By increasing the sintering temperature to 1450 °C, similar results were observed (Figure 2c,d), but both surfaces were much denser than the sample sintered at 1400 °C, leaving much fewer pores on the surfaces. 

### 2.2. Mechanical Property

**Figure 3:**
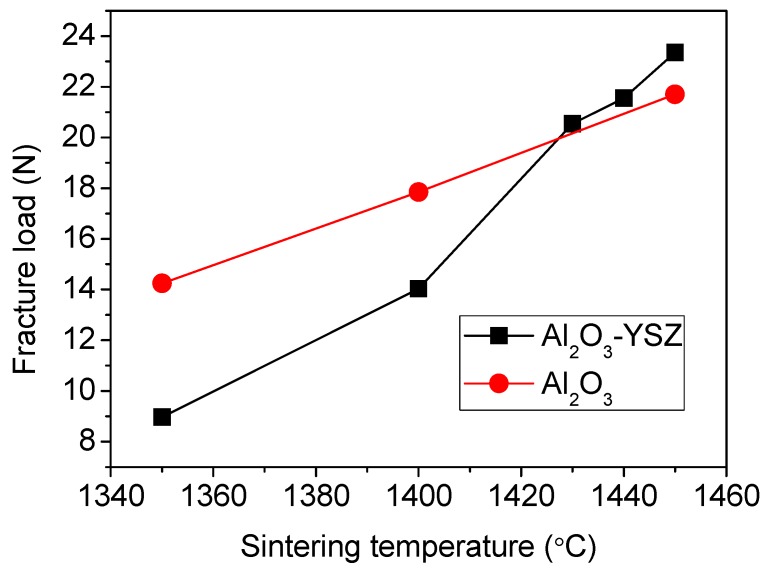
Three point bending fracture load of the Al_2_O_3_-YSZ and pure alumina membranes [8].

The sintering temperature affects the mechanical property, as expected. Figure 3 gives three-point bending results of the reinforced Al_2_O_3_-YSZ capillary membranes. The results are similar to those of pure alumina seven-channel capillary tubes [8]. It is interesting to note that at a sintering temperature lower than 1430 °C, the fracture load of the Al_2_O_3_-YSZ tube is in fact lower than the pure alumina tube, but it is increased quickly as the sintering temperature is increased. This behavior indicates a change in the sintering mechanism that might occur when the Al_2_O_3_-YSZ tube was sintered at a temperature higher than 1430 °C. The change in sintering mechanisms at higher sintering temperatures is also reflected by the pore structure, which will be discussed later. 

**Figure 4:**
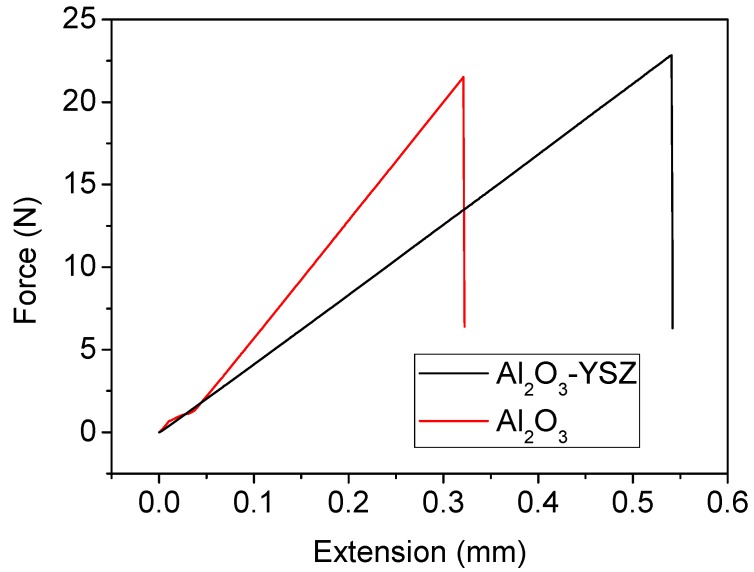
Extension-force bending curves of the Al_2_O_3_-YSZ and pure alumina seven-channel capillary membranes.

Figure 4 shows force-extension curves of the Al_2_O_3_-YSZ seven-channel capillary tube sintered at 1440 °C and a pure alumina seven-channel tube sintered at 1450 °C. Although these two tubes have very close fracture loads (22.85 N for Al_2_O_3_-YSZ *vs.* 21.52 N for pure alumina), their bending extensions at the breaking point are quite different. The Al_2_O_3_-YSZ tube could extend 68.3% more than the pure alumina tube, meaning that it can adsorb 78.7% more energy than the pure alumina tube before breaking if the tube undergoes a continuous impact. In addition, if the tubes are subject to pulsed impacts where the actual force produced is affected by the initial momentum and the acting time, the Al_2_O_3_-YSZ tube would be more advantageous, because it can prolong the acting time and thus help to reduce the actual force on it.

### 2.3. Pore Size and Water Permeation

**Figure 5:**
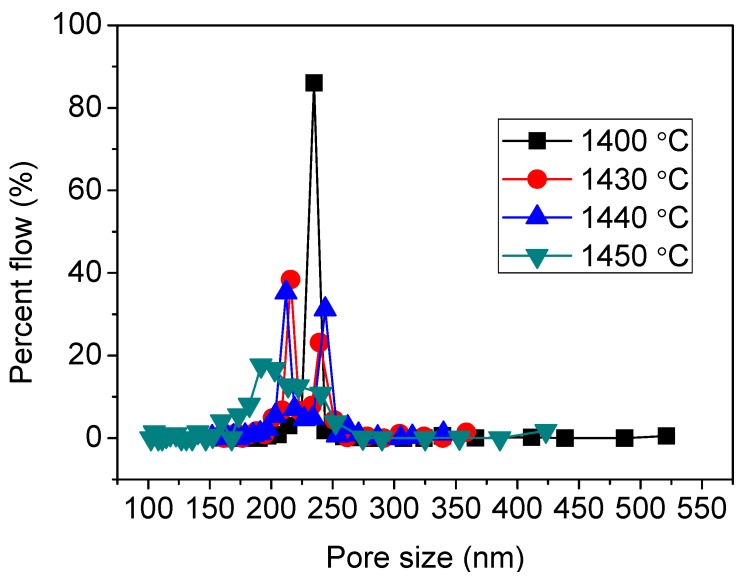
Gas-liquid-displacement pore size distributions of the Al_2_O_3_-YSZ membranes sintered at different temperatures.

The size of open pores and their size distribution of the Al_2_O_3_-YSZ membranes sintered at 1400–1450 °C were measured by using the gas-liquid displacement method. The mean flow pore size of all samples is in the range of 210–240 nm, which is not significantly affected by the sintering temperature. However, the pore size distribution (Figure 5) shows a turning from a singular distribution to a binary distribution when the sintering temperature is elevated from 1400 to above 1430 °C. For the sample sintered at 1400 °C, only one peak at 235 nm with a percent flow of 86% can be seen, *i.e.*, a very narrow pore size distribution. However, for the sample sintered at 1430 °C, two peaks at 240 and 216 nm are observed. Similarly, for the sample sintered at 1440 °C, the two peaks are at 244 and 212 nm. For the sample sintered at 1450 °C, the two peaks merge into a broad peak, and the mean flow pore size shifts to 215 nm. The change in the pore size distribution is concurrent with the change in mechanical property, corresponding a subtle change of the sintering behavior. 

**Figure 6:**
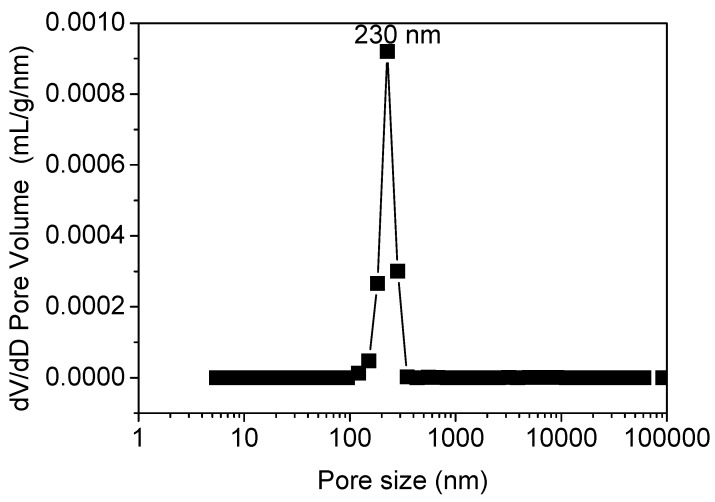
Mercury intrusion porosimetry result of the Al_2_O_3_-YSZ membrane sintered at 1440 °C.

The pore size distribution inside the sample sintered at 1440 °C was also determined by mercury porosimetry, as shown in Figure 6. The mercury porosimetry result shows a single narrow peak at 230 nm, identical to the mean flow pore size determined by the gas-liquid displacement. This result implies that the effective transport path in this membrane is the inter-granule pores in the sponge-like region, which is not affected by the micro-channels present in the membrane. The binary distribution of the pore structure obtained by the gas-liquid displacement was not observed when using mercury porosimetry, which is likely due to the natural difference between these two methods, *i.e.*, mercury porosimetry measures all pores including dead-ended pores, whereas gas-liquid displacement only measures open pores.

**Figure 7:**
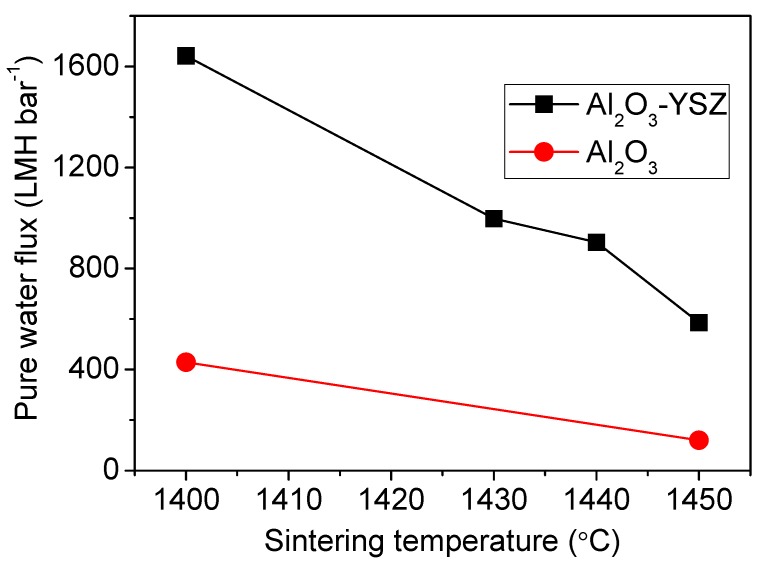
Pure water fluxes of the Al_2_O_3_-YSZ membranes. Water fluxes of a pure alumina membrane with same structure [8] are shown for comparison.

Unsurprisingly, pure water flux of the Al_2_O_3_-YSZ seven-channel membrane declines with increasing sintering temperature (Figure 7). The water flux dropped from 1642 to 586 LMH/bar when the sintering temperature was increased from 1400 to 1450 °C. The pure water flux is very sensitive to the sintering temperature at a range between 1430–1450 °C, which was 997 LMH/bar when sintered at 1430 °C and 904 LMH/bar when sintered at 1440 °C. They were considerably higher than the value of the 1450 °C sample, while the fracture load didn’t show significant changes. As a comparison, pure alumina seven-channel tubes showed much lower permeation rates, which was only 429 LMH/bar when sintered at 1400 °C and 120 LMH/bar when sintered at 1450 °C [8]. Therefore, for the Al_2_O_3_-YSZ membranes, the best sintering temperature is 1430–1440 °C, at which both excellent mechanical property and good permeation characteristics can be obtained.

## 3. Experimental Section 

### 3.1. Materials

Commercial aluminium oxide (Al_2_O_3_) (alpha phase, 99.9% metals basis, surface area 6–8 m^2^/g, d_50_ = 1 µm, Alfa-Aesar) and 3-YSZ (Inframat®, d_50_ < 0.5 μm, Inframat Corporation) were used as supplied. Polyethersulfone (PESf) (Radal A300, Ameco Performance, USA) was used as polymeric binder. Dimethyl sulphoxide (DMSO, HPLC grade, VWR) was used as solvent. Arlacel P135 (polyethylene glycol 30-dipolyhydroxystearate, Uniqema) is used as additive. De-ionized water was used as the external and internal coagulants.

### 3.2. Fabrication of Seven-Channel Al_2_O_3_-YSZ Capillary Membranes

The fabrication process is based on the combined phase-inversion and sintering technique. Generally, a uniform suspension composed of ceramic particles, solvent and polymeric binder, as well as an additive acting as a dispersant, was prepared through ball milling. The suspension was then degassed with stirring to remove bubbles, prior to being transferred into a 200 ml stainless steel syringe that is controlled by a syringe pump (Harvard PHD22/200 HPsi, Harvard Apparatus, Holliston, MA, USA). The suspension can thus be extruded through a seven-channel spinneret into the external coagulation bath. Meanwhile, DI water used as the internal coagulant was introduced through the spinneret with another syringe pump of the same model. The precursor capillary tubes were removed from the external coagulant bath when the phase inversion was completed, and were then dried and straightened at room temperature, and cut into a required length for the subsequent high temperature sintering. Details of the fabrication process are listed in Table 1. The tubes were then sintered in a tubular furnace for five hours to remove the organic materials and gain strength.

**Table 1:** Fabricating parameters of the seven-channel Al_2_O_3_-YSZ capillary membrane.

Suspension Composition					Fabrication		
Alumina (wt.%)	YSZ (wt.%)	DMSO (wt.%)	PESf (wt.%)	Additive (wt.%)	Extrusion rate (mL/min)	Channel liquid rate (mL/min)	Air gap (cm)
38.3	24.8	29.2	7.3	0.4	5.3	12	2

### 3.3. Characterizations

Morphologies and microstructures were characterized by using a scanning electron microscope (SEM, LEO Gemini 1525 FEGSEM). Pore size and pore size distribution were determined by using gas-liquid displacement measurements with PoroLux 1000 Porometer and mercury intrusion porosimetry with Micromeritics Autopore IV. Mechanical property was evaluated by using an Instron materials testing system (Model 5544) with a 5 kN load cell, using a three-point bending method, where the samples were positioned onto a sample holder with a span of 60 mm. Pure water permeation of the seven-channel capillary tubular membranes was evaluated by using dead-end filtration testing method under the out-to-in mode after being sealed into a stainless vessel using epoxy resin. Because the multi-channel tubes were used as single-channel membranes, the outer surface area, instead of the actual permeation area, was used for calculating all the permeation fluxes to achieve straightforward comparisons.

## 4. Conclusions 

YSZ reinforced alumina seven-channel capillary membranes were prepared by using the combined phase-inversion and sintering method. The addition of YSZ can effectively increase the extension under an impact and also improve the fracture load of the capillary membrane. At sintering temperatures of above 1430 °C, the Al_2_O_3_-YSZ membranes showed a change in trend of the mechanical property and different pore size distributions, due to the changes in the sintering behavior. The YSZ reinforced alumina membranes showed substantially improved water permeation fluxes over pure alumina membranes with similar pore sizes. The YSZ reinforcedcapillary membranes showed good potential to meet the requirements for industrial applications.

## References

[1] Li K. (2007). Ceramic Membranes for Separation and Reaction.

[2] Kingsbury B.F.K., Li K. (2009). A morphological study of ceramic hollow fibre membranes. J. Membr. Sci..

[3] Kingsbury B.F.K., Wu Z., Li K. (2010). A morphological study of ceramic hollow fibre membranes: A perspective on multifunctional catalytic membrane reactors. Catal. Today.

[4] Wu Z., Wang B., Li K. (2010). A novel dual-layer ceramic hollow fibre membrane reactor for methane conversion. J. Membr. Sci..

[5] Kilgus M., Gepert V., Dinges N., Merten C., Eigenberger G., Schiestel T. (2006). Palladium coated ceramic hollow fibre membranes for hydrogen separation. Desalination.

[6] Wei C.C., Chen O.Y., Liu Y., Li K. (2008). Ceramic asymmetric hollow fibre membranes—One step fabrication process. J. Membr. Sci..

[7] Kanawka K., Othman M.H.D., Wu Z., Droushiotis N., Kelsall G., Li K. (2011). A dual layer Ni/Ni-YSZ hollow fibre for micro-tubular SOFC anode support with a current collector. Electrochem. Commun..

[8] Lee M., Wu Z., Wang B., Li K. (2015). Micro-structured alumina multi-channel capillary tubes and monoliths. J. Membr. Sci..

[9] Wang J., Stevens R. (1989). Zirconia-toughened alumina (ZTA) ceramics. J. Mater. Sci..

[10] Lee M., Wang B., Wu Z., Li K. (2015). Formation of micro-channels in ceramic membranes—Spatial structure, simulation, and potential use in water treatment. J. Membr. Sci..

